# The prone position must accommodate changes in IAP in traumatic brain injury patients

**DOI:** 10.1186/s13054-021-03506-8

**Published:** 2021-04-07

**Authors:** Wojciech Dabrowski, Dorota Siwicka-Gieroba, Chiara Robba, Rafael Badenes, Manu L. N. G. Malbrain

**Affiliations:** 1 Department of Anesthesiology and Intensive Care, Medical University of Lublin, 20-954 Lublin, Poland; 2 Department of Anaesthesia and Intensive Care, Policlinico San Martino, Genoa, Italy; 3 Department of Anesthesiology and Intensive Care, Hospital Clìnico Universitario de Valencia, University of Valencia, Valencia, Spain; 4 Department of Electronics and Informatics (ETRO), Faculty of Engineering, Vrije Universiteit Brussel (VUB), Brussels, Belgium; 5 International Fluid Academy, Lovenjoel, Belgium

Dear Editor,

Recently, Bernon et al. evaluated in a retrospective study the safety and efficacy of prone position (PP) in patients treated for traumatic brain injury (TBI) and moderate-to-severe acute respiratory distress syndrome (ARDS) [1]. They analyzed changes in P_a_O_2_/FiO_2_ and intracranial pressure in 10 patients during PP. Although PaO_2_/FiO_2_ improved, PP was discontinued due to a raised intracranial pressure (ICP) in 50% of patients. Additionally, they found that all patients with ICP > 17.5 mmHg and 28% of patients with ICP < 17.5 mmHg prior PP had intracranial hypertension (ICH, defined as one or more ICP elevations > 25 mmHg) following PP. They concluded that monitoring of the brain compliance, ICP and the tolerance to venous return obstruction (Queckenstedt’s maneuver) could be useful before decision of PP.

Severe ARDS makes the ventilator management of patients with TBI even more challenging. The European Society of Intensive Care Medicine strongly recommends to consider PP in patients with concomitant ARDS and TBI, if ICP is stable [2]. When PP is necessary, clinicians suggest to strictly monitor ICP, possibly with a multimodal neuromonitoring approach [1, 3] to early and promptly treat neurological complications. However, PP may increase intracranial pressure (ICP) via a reduction of blood outflow from the brain.

Several factors may impair venous outflow from the brain, and elevated intra-abdominal pressure (IAP) is one of them. Significant increase in IAP closely corresponds to an increase in central venous pressure, jugular venous bulb pressure and low jugular venous bulb saturation in critically ill patients [4]. It was documented that increased IAP played an important role in developing intracranial complications during neurosurgical procedures in patients suffering from idiopathic ICH, TBI and during hydrocephalus therapy [5]. An incorrect PP can therefore increase intra-thoracic pressure via diaphragm elevation, causing impaired blood outflow from the brain leading to increase in ICP (Fig. 1). Hence, the elevated IAP following abdominal compression during PP plays a crucial role during ICP management, particularly in obese patients. Although we agree with the suggestions from Bernon et al. [1] regarding the need to close brain-monitoring in ARDS patients with TBI undergoing PP, we further suggest to include IAP monitoring and to carefully check the patient’s position in order to avoid abdominal compression during PP. Further studies should be performed to explain the relationships between changes in IAP and risk of increase in ICP in patients with concomitant ARDS and TBI treated with PP.

**Fig. 1:**
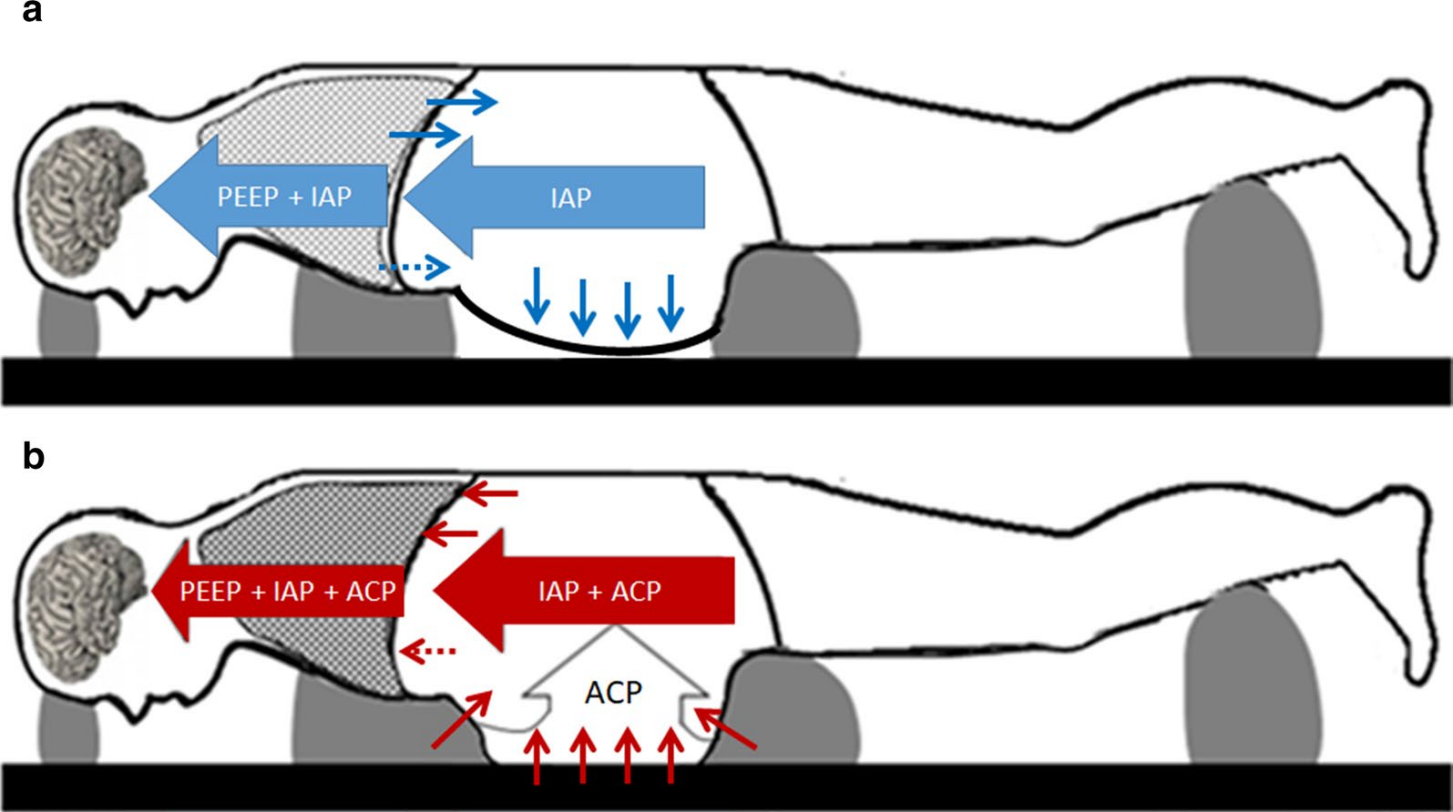
Diagram illustration correct (**a**) and incorrect (**b**) prone positioning in a patient treated for traumatic brain injury complicated with moderate-to-severe acute respiratory distress syndrome (ARDS). Correct positioning with abdominal suspension, so that the abdomen can hang free will not increase IAP during PP. An incorrect positioning on the contrary will increase IAP by a back pressure resulting from compression of the abdomen by the bed and faulty suspension. *IAP* intra-abdominal pressure, *ACP* abdominal compression pressure, *PEEP* positive end-respiratory pressure

## Data Availability

Not applicable.
