# Supplementary material for: The prone position must accommodate changes in IAP in traumatic brain injury patients
Source: Crit Care. 2021 Apr 7;25:132. doi: 10.1186/s13054-021-03506-8 (PMC8024931; doi:10.1186/s13054-021-03506-8)
Supplement: Related texts

# Reply: Prone positioning modalities are essential for good tolerance in patients with acute brain injury

**Authors:** Pauline Bernon, Anne-Claire Lukaszewicz, Baptiste Balança

**Affiliations:** 6 grid.414243.4 0000 0004 0597 9318 Hospices Civils de Lyon, Département d’anesthésie réanimation, Hôpital Neurologique Pierre Wertheimer, 59 Boulevard Pinel, 69500 Bron, France; 7 grid.412180.e 0000 0001 2198 4166 EA 7426 PI3 (Pathophysiology of Injury-induced Immunosuppression), Hospices Civils de Lyon/Université Claude Bernard Lyon 1/bioMérieux, Hôpital E. Herriot, 5, place d’Arsonval, 69437 Lyon, Cedex 03, France; 8 grid.461862.f 0000 0004 0614 7222 U1028, UMR 5292, Lyon Neuroscience Research Center, Bron, France

Dear Editor,

Dabrowski et al. published a letter in the journal about our work reporting data on the safety and efficacy of prone positioning (PP) in patients with acute brain injury and moderate-to-severe acute respiratory distress syndrome (ARDS) [1]. They suggest monitoring intra-abdominal pressure (IAP) in addition to brain compliance to evaluate the risk of impaired brain venous return during PP particularly in obese patients. In our cohort, intracranial hypertension (ICH, intracranial pressure-ICP > 25 mmHg) occurred in 14/27 patients (52%) leading to discontinue PP in 5/27 patients. Only 7/27 patients (26%) had a body mass index over 30 kg/m^2^, 30% of whom (*n* = 3) had ICH during PP. The number of obese patients at risk of increased IAP was too small to conclude, and we agree with Dabrowski et al. that IAP plays a critical role in venous return changes during PP. The pressure in the right atrium, which drives cerebral blood outflow, is influence by cardiac output, intrathoracic pressure and to some extent by the venous pressure from other organs. PP is expected to improve thoracic compliance and thereby venous return; however, the increase in IAP may hinder this beneficial effect. For instance, during elective spine surgery the only frame that can reduce hemodynamic changes triggered by PP allows the abdomen to hang free [6] thereby preventing IAP elevation. This strategy has also been implemented in the intensive care unit with circular pillow during PP [7]. IAP release may therefore be transmitted to the right atrium and eventually to the brain, thereby improving cerebral outflow. Consequently, monitoring IAP could allow optimizing the positioning of patients with a high central venous pressure. In our opinion, head position remains one major determinant of cerebral blood outflow. It must remain on the axis in order to avoid compression of the jugular veins. The elevation of the head above the heart level is also important and allows to reduce intracranial pressure (ICP) in brain injured patients [8]. We agree that abdominal pressure release and monitoring should be considered during PP, but we would argue to test the tolerance to an obstacle to cerebral blood outflow before PP to identify patient at risk of IH. The evaluation of a protocol that standardize head position and abdomen release is therefore necessary to decrease the risk of IH during PP sessions in patients with acute brain injury and ARDS.
